# Giant Arteriovenous Malformation of the Neck

**DOI:** 10.1155/2015/124010

**Published:** 2015-08-10

**Authors:** P. A. Dieng, P. S. Ba, M. Gaye, S. Diatta, M. S. Diop, E. Sene, A. G. Ciss, A. Ndiaye, M. Ndiaye

**Affiliations:** Service de Chirurgie Thoracique et Cardiovasculaire, Centre Hospitalier National Universitaire de Fann, Dakar, Senegal

## Abstract

Arteriovenous malformations (AVM) have a wide range of clinical presentations. Operative bleeding is one of the most hazardous complications in the surgical management of high-flow vascular malformations. In the cervical region, the presence of vital vascular structures, such as the carotid artery and jugular vein, may increase this risk. This is a case of massive arteriovenous malformation deforming the neck and the face aspect of this aged lady and growing for several years. A giant mass of the left neck occupied the carotid region and the subclavian region. The AVM was developed between the carotid arteries, jugular veins, and vertebral and subclavian vessels, with arterial and venous flux. The patient underwent surgery twice for the cure of that AVM. The first step was the ligation of the external carotid. Seven days later, the excision of the mass was done. In postoperative period the patient presented a peripheral facial paralysis which completely decreased within 10 days. The first ligation of the external carotid reduces significantly the blood flow into the AVM. It permitted secondarily the complete ablation of the AVM without major bleeding even though multiple ligations were done.

## 1. Introduction

Arteriovenous malformations (AVM) are part of the big chapter of vascular anomalies. They have a wide range of clinical presentations and an unpredictable course [1]. Traumatism is the most common cause of arteriovenous communications between the blood vessels in the cervical area. Spontaneous malformations in this area also occur [2].

Intraoperative bleeding is one of the most hazardous complications in the surgical management of high-flow vascular malformations. It is even more relevant for massive AVM within the cervical region, where the presence of vital vascular structures, such as the carotid artery and jugular vein, may evolve in uncontrollable bleeding [3]. The care of congenital AVM arteriovenous malformations is challenging.

This is a case of massive arteriovenous malformation deforming the neck and the face aspect of this aged lady and growing for several years.

## 2. Case Report

A 56-year-old woman presented with a left cervical mass growing for 3 years. In her medical history we did not find traumatism but hypertension.

**Figure 1:**
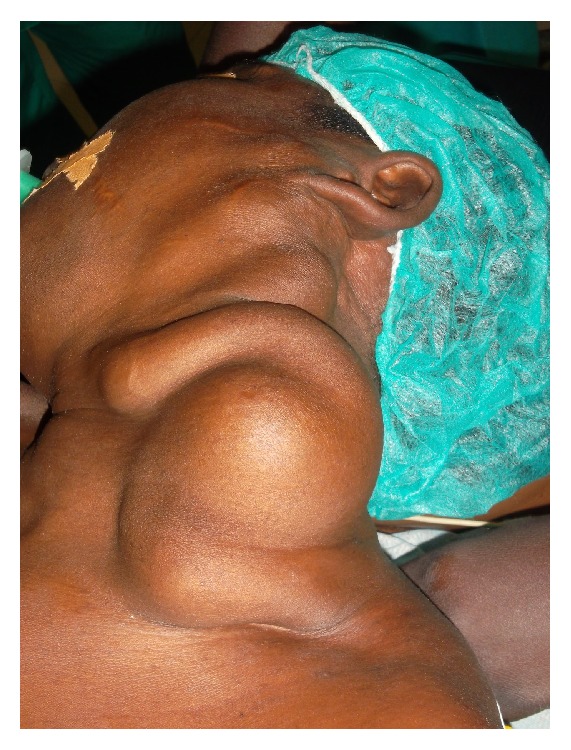
Anterolateral view of the cervical AVM.

The physical examination showed a giant mass of the left neck occupying the carotid region and the subclavian region, measuring 20 cm × 15 cm × 10 cm (Figure 1). The vascular characters with thrill and systolic-diastolic bruit were observed all over the neck.

The Doppler ultrasound confirmed an arteriovenous malformation in the neck between the carotid arteries and jugular veins with arterial and venous flux.

**Figure 2:**
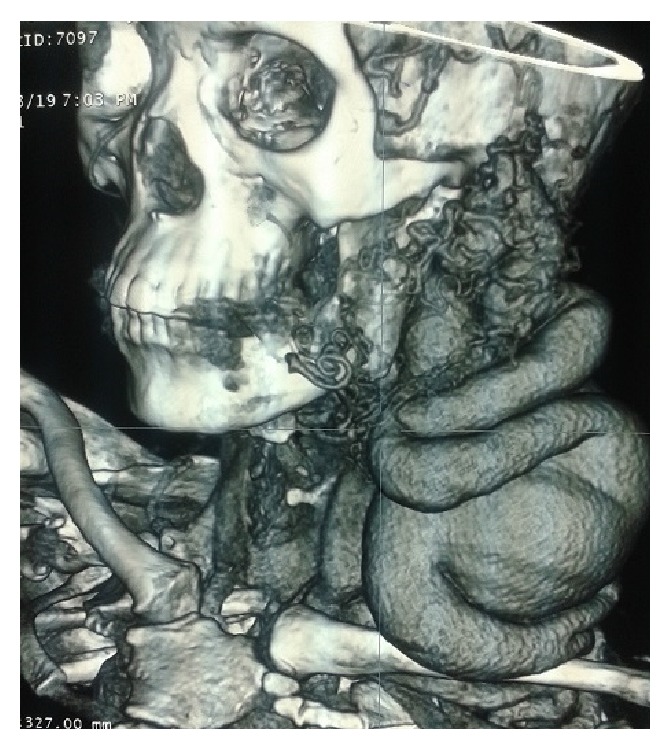
CT scan image of the cervical AVM. Anterolateral view showing connections with carotid, jugular, and subclavian.

**Figure 3:**
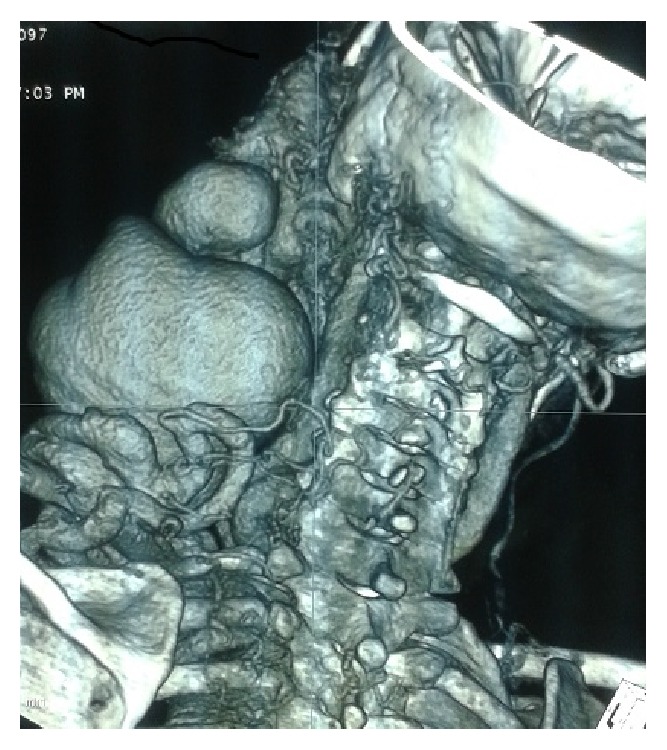
CT scan. Posterior view of cervical AVM showing connections with vertebral.

**Figure 4:**
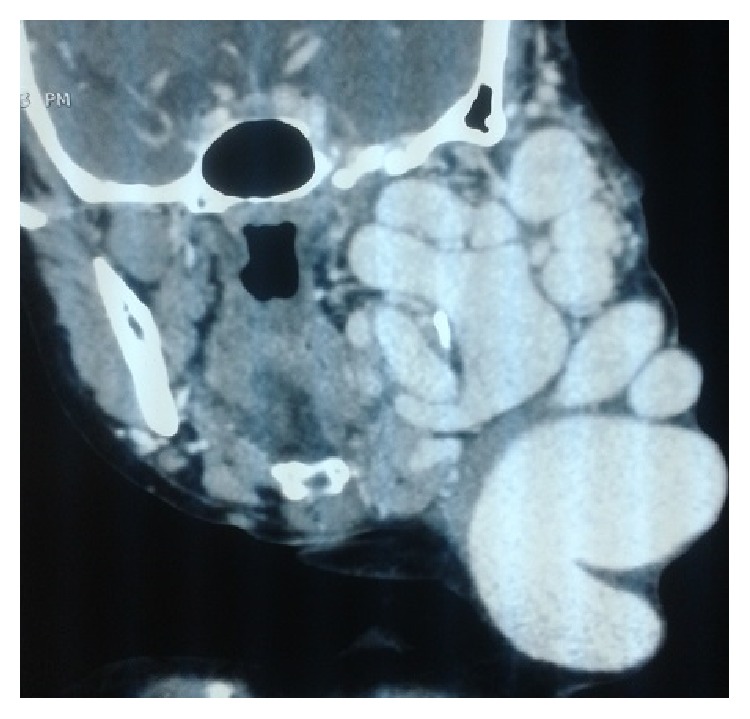
Scan reconstruction showing the contrast filing the cervical AVM.

The CT scan showed a tortuous vascular mass measuring 15 × 10 cm (Figure 2). The venous drainage came from innominate venous trunk. The arterial source came from the external carotid artery (Figures 3 and 4). Besides that CT showed an arteria lusoria.

**Figure 5:**
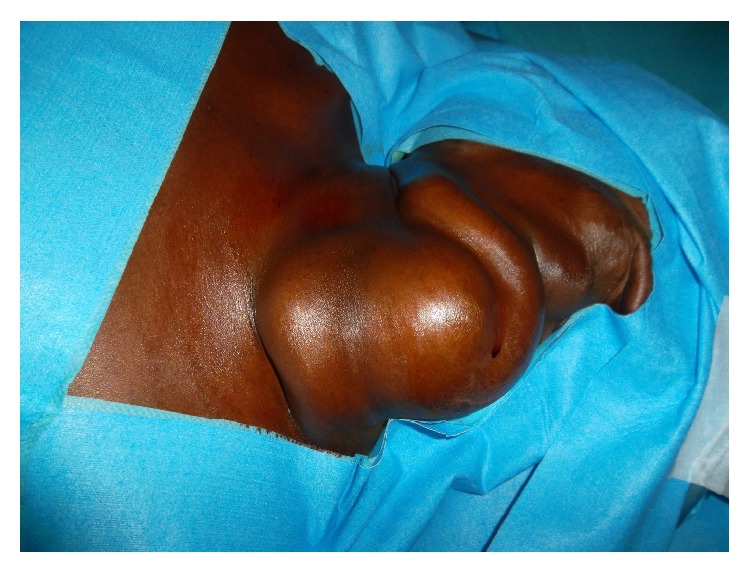
Preoperative view of the AVM exposed before surgical access.

The patient underwent surgery twice for the cure of that AVM (Figure 5). The first step was the ligation of the external carotid. This allowed regression of the bruit and a significant drop of the arterial flux in the mass.

**Figure 6:**
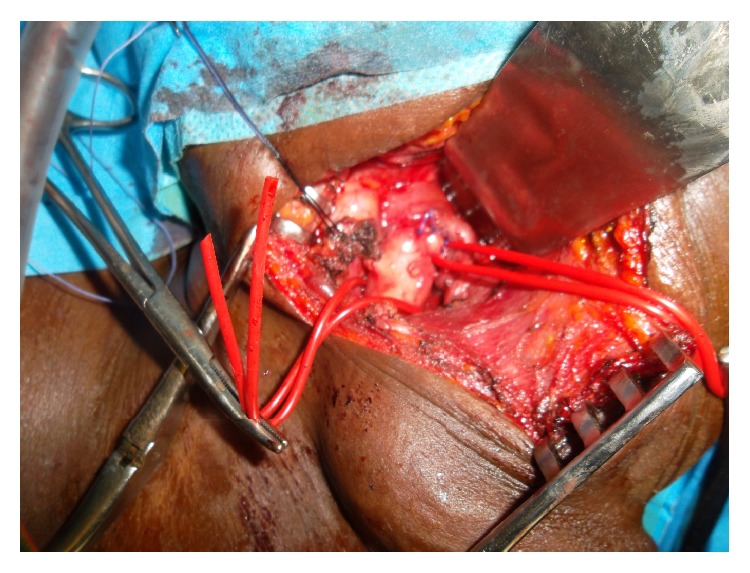
Operative view of the external carotid artery connected to AVM.

Seven days later, the excision of the mass was scheduled. The arteriovenous malformation was exposed over left longitudinal cervicotomy. The left carotids were dissected as well the jugular vein which was dilated and aneurysmal. More than 50 vessels were identified and ligated. Some of them measured more than 3 mm. They joined the mass to the left external carotid, the external and internal jugular veins, the left subclavian artery and vein, and the left vertebral artery and vein. After these vessels ligation, the mass was completely excised without major bleeding (Figure 6). Hemodynamic status did not need a blood transfusion.

In postoperative period the patient presented a peripheral facial paralysis which completely decreased within 10 days after corticoids therapy.

## 3. Discussion

The localization of arteriovenous malformation on the neck induces surgical difficulties. The complete excision of the mass without nerve or vascular injury or major bleeding is a surgical challenge. Because of the risk of bleeding, some authors indicate proceeding with 2 steps for the surgical care [1, 4]. To reduce the blood flow into the mass, embolization is used for some surgical teams [3].

Embolization is done before surgery to reduce the inflow, to permit formation of thrombus before the resection. Some authors describe the use of embolization at the same time of surgery. Some teams use surgery only after embolization failed. Considering the high flow of this kind of fistula, embolic materials such as gel foam and ethanol are at higher risk for pulmonary embolism [5].

The first step could be an arterial ligation as we had done in this case. Imaging permits identifying the inflow vessels. It could be one artery, but in complex arteriovenous malformation there are several inflow vessels [6]. In this case there are more than 50 vessels connected to the mass; most of them came from external carotid artery. But they came also from vertebral vessels, subclavian vessels, and vertebral vessels. The main risk of surgery was bleeding due to the gigantism of the mass and the complexity of this AVM. The first ligation of the external carotid reduces significantly the blood flow into the AVM. That first procedure permitted secondarily the complete ablation of the AVM without major bleeding even though multiple ligations were done.

The external carotid ligation is a controversial technique, because of risk of recurrence. For Porcu, the ligation of the feeding arteries is ineffective or can offer only a temporary improvement because of the recruitment of distal vasculature with persistence of the fistula [7]. In this case, authors used it just as the first step to prevent major bleeding, and it was followed by complete ablation of the mass.

The resection should be as complete as possible because recurrence rate is high [4, 8]. The recurrence of the mass makes the redo intervention very difficult because of fibrosis and modification of the anatomy of the neck.

Primary surgical correction by ligation and resection give good results in very selective cases with single communication [8]. Facial nerve injury has, however, been reported [9, 10]. In this case the transient facial paralysis was due to inflammation and edema and regressed under medical treatment.

Traumatism is a frequent cause of arteriovenous malformations, but most of the time the AVMs are congenital [2]. It is the case for this lady; even though it appears in the adult age, the appearance is congenital malformation with multiples fistulas.

The gigantism of the cervical mass induces cosmetic disfigurement, with social impact. That disgracious appearance induces sometimes psychiatric issue [4].

## 4. Conclusion

Surgery of cervical arteriovenous malformation is challenging. The mass was linked to multiple arteries and veins. The gigantism and complexity of this AVM put the patient at risk of injury. Primary arterial ligation reduces significantly the flow and permits secondarily the mass resection without major bleeding.
